# Gastroprotective Effects of Fermented Lotus Root against Ethanol/HCl-Induced Gastric Mucosal Acute Toxicity in Rats

**DOI:** 10.3390/nu12030808

**Published:** 2020-03-19

**Authors:** Jeong-Hyun Yoo, Eun-Jung Park, So Hyeun Kim, Hae-Jeung Lee

**Affiliations:** 1 Institute for Aging and Clinical Nutrition Research, Gachon University, Gyeonggi-do 13120, Korea; yjh8252@naver.com (J.-H.Y.); ejpark@gachon.ac.kr (E.-J.P.); 2 Department of Food and Nutrition, Gachon University, Gyeonggi-do 13120, Korea; sohun94@naver.com

**Keywords:** fermented lotus root, gastric ulcer, gastroprotective effect, anti-inflammatory effect, antioxidant effect

## Abstract

Gastric ulcers are a common gastrointestinal disease across the globe. Alcohol consumption is the primary cause of gastric carcinogenesis and progression. We investigated the gastroprotective effects of fermented lotus root (FL) against ethanol (EtOH)/HCl-induced gastric ulcers in a rat model and the conceivable underlying mechanisms involved. Rats received different doses of FL (50, 100, and 200 mg/kg) or ranitidine (positive control, 30 mg/kg) via oral gavage daily for 14 days. One hour after the last oral administration of FL, the EtOH/HCl mixture was orally intubated to induce gastric damage. Oral administration of FL significantly alleviated the gastric lesions. Moreover, FL also elevated the amounts of nitric oxide and the antioxidant enzyme activities of superoxide dismutase, glutathione peroxidase, and catalase in the stomach. To verify the gastric mucosal defense mechanism, inflammation-related genes were measured. Our results revealed that FL effectively inhibited gastric mucosal damage via downregulation of the nuclear factor-kappaB (NF-κB) response in the stomach. The administration of FL significantly lowered the gastric mRNA expression of inflammation-related genes, including *NF*-*κb1*, tumor necrosis factor-*α*, interferon γ, and prostaglandin-endoperoxide synthase 2, compared with the gastric ulcer control group. In addition, the NF-κB signaling pathway-related protein markers inhibitor of κB (IκB)-α, IκB kinase, and NF-κB were significantly reduced in the FL groups. Taken together, these data suggest that FL administration may have potential as an alternative treatment for gastric ulcers due to its antioxidant and anti-inflammatory effects and its ability to promote the recovery of gastric mucosa.

## 1. Introduction

Gastric ulcers are the most common multifactorial gastrointestinal disorder and are accompanied by symptoms such as bloating, gas, fever, abdominal pain, nausea, and vomiting. Major causes include infection with the bacterium *Helicobacter pylori*, (*H. pylori*), long-term use of aspirin and nonsteroidal anti-inflammatory drugs (NSAIDs), smoking, and alcohol consumption [1]. These risk factors can erode the stomach’s defense system against the overproduction of acid, and the damage to the stomach wall can result in an ulcer. Furthermore, excessive alcohol intake can directly affect the gastric mucosal tissues and increase the permeability by an increase in acid back diffusion. This disruption of the mucosal defensive and aggressive factors may result in gastric cancer [1]. 

The gastric mucosal barrier protects the stomach wall from local damage and maintains the structural and functional mucosal integrity through the mucosal defense mechanisms [2]. When the mucosal defense is weakened or overwhelmed by injurious factors in the stomach, the gastric mucosal barrier allows acid to diffuse back into the mucus layer, where it can cause damage to the stomach itself. The gastric mucosal integrity is maintained by pre-epithelial (mucus, bicarbonate, and phospholipids), epithelial (prostaglandins, heat shock proteins, trefoil factor family peptides (TFFs), and cathelicidins), and post-epithelial (mucosal blood flow, such as nitric oxide (NO) and prostacyclin) factors [3].

Oxidative stress and stomach disorders are closely related pathophysiological processes [4]. The over-production of reactive oxygen species (ROS) under oxidative stress results in cellular damage. To maintain gastrointestinal homeostasis through the scavenging of ROS, gastric cells have several endogenous antioxidant enzymes, such as superoxide dismutase (SOD), glutathione peroxidase (GPx), and catalase (CAT). SOD catalyzes the dismutation of O_2_^−^ into H_2_O_2_ [5]. Reduced SOD activity causes gastric ulcers, and increased SOD activity has been associated with ulcer healing in patients [6]. H_2_O_2_ that is formed is further broken down by antioxidant enzymes, GPx or CAT [7]. GPx reduces H_2_O_2_ and inhibits lipid peroxidation directly or indirectly by the mediation of lipid peroxides. CAT accelerates the H_2_O_2_ breakdown to water and oxygen. Therefore, the antioxidant defense system has an important role in the protection of the gastric mucosal integrity.

Nuclear factor-kappa B (NF-κB) is a transcription factor that regulates the expression of many genes responsible for inflammation. NF-κB can be activated by oxidative stress [8,9]. The activation of NF-κB involves the phosphorylation of the inhibitor of κB (IκB) kinases (IKK) complex. This complex phosphorylates the IκB molecules and results in the proteasomal degradation of IκB and the nuclear translocation of NF-κB, leading to the expression of inflammation-related markers. 

*Nelumbo nucifera* Gaertn (*N. nucifera*) is in the family *Nelumbonaceae*. *N. nucifera* is a perennial water plant that grows in Korea, India, China, Japan, and Siberia. Its various parts (roots, leaves, and flowers) have been used as a herbal medicine for about 7000 years in Asia [10,11]. *N. nucifera* (lotus root) is commonly consumed and referred to as “yeongeun (연근)” in Korea, “kamal kakri” in India, “Lián ǒu (蓮藕)” in China, and “renkon (れんこん)” in Japan. Particularly, the use of lotus root is common as a traditional medicinal food in Korea based on Donguibogam, a Korean traditional medical encyclopedia, written by Jun Heo [12]. It was reported that *N. nucifera* has a variety of biological activities, including antioxidant, antiproliferative, hepatoprotection, and anti-inflammatory effects [13,14,15,16]. Although lotus plants are popular in Asia, there are few reports on lotus root research. 

To the best of our knowledge, this is the first study to investigate the protective effects of lotus root in a gastric mucosal damage rat model. An alcohol-induced gastric ulcer model has been commonly used to study both pathogenesis and therapies for human ulcerative disease [17,18,19]. This is an effective experimental model to evaluate the gastroprotection of a tested compound. The present study investigated the anti-ulcer properties of fermented lotus root in experimental gastric ulcers induced by 60% ethanol (EtOH)/150 mM HCl in rats.

## 2. Materials and Methods

### 2.1. Preparations of Fermented Lotus Root

Fermented lotus root (FL) powder was prepared by standard production processes and supplied by Hwashin Farming Corporation (Gyeongsangnam-do, Korea). Briefly, the lotus root was washed to remove impurities. Next, the lotus root and natural plant extracts (lotus root 7%, lotus leaf 60%, jujube 32.8%, and Korean Panax ginseng 0.2%) were mixed and fermented with *Saccharomyces cerevisiae*. After steaming and drying for 6 days, the lotus root was dried at 37.5 °C and further incubated at 47.6 °C for 15 days. Last, the fermented lotus roots were ground and used in the experimental dietary preparation. The FL powder contained 3.01 mg of total flavonoid, 11.1 mg of total polyphenol, and 1.53 mg of linoleic acid per gram. The powdered fermented lotus root was stored at − 20 °C until use.

### 2.2. Animals and Experimental Protocol

Male Sprague Dawley (SD) rats at the age of 6 weeks were purchased from Orient Bio Co. Ltd. (Gyeonggi-do, Korea). A total of 42 rats were kept in cages with food and water ad libitum, in a controlled temperature (20–25 °C), controlled humidity (50–55%), and with a 12-h light-dark cycle. The rats were randomly divided into six groups (*n* = 7 for each group): normal control (NC), gastric ulcer model control (HE), positive control (PC; ranitidine 30 mg/kg), and fermented lotus root groups with different doses (50, 100, and 200 mg/kg; LF, MF, and HF, respectively). The animals received a standard rodent chow (Teklad Global 18% Protein Rodent Diet 2018S; Harlan Laboratories INC., USA). 

The normal control and model control groups were treated with an equal volume of deionized water by oral gavage. Ranitidine (Sigma-Aldrich, St. Louis, MO, USA) was used as a reference gastroprotective drug and H_2_ histamine receptor antagonist [17,20]. The rats in the treatment group were orally administered 1 mL of the test substances (ranitidine or fermented lotus root) dissolved in distilled water, consecutively for 14 days. On the last day of treatment, all animals were deprived of food for 24 h overnight in a cage with wide-mesh wire bottoms to prevent coprophagia. The experimental groups were fed orally with the EtOH/HCl mixture (1 mL/60% EtOH containing 150 mM HCl) to induce gastric mucosal damage [18], while those in the normal control groups were orally administered with an equal volume of distilled water. One hour after induction, the rats were sacrificed using CO_2_. 

All animals were maintained and used in accordance with the guidelines issued by Gachon University for the care and use of laboratory animals (approval number GIAUAC–R2018010).

### 2.3. Preparation of the Stomach Tissue Sample and Determination of the Gastric Lesion Index

The rat stomachs were immediately removed and dissected along the greater curvatures at sacrifice. The stomachs were rinsed with cold phosphate-buffered saline (PBS) and the gastric tissues were photographed. The gastric damage (erosion or ulcer) area was determined with “Image J” image processing software (NIH, USA). The lesion index and lesion inhibition rate for each rat was calculated with the following formula (Equations (1) and (2)):

lesion index (%) = (gastric damage area of each rat/gastric mucosal area of each rat) × 100(1)

lesion inhibition rate (%) = (1−total gastric damage area of sample treated group (PC and FL groups)/total gastric damage area of the gastric damage control (HE group) × 100.(2)

After taking photographs, small pieces of the stomach were immediately stored in liquid nitrogen for the determination of biomarkers, reverse transcription real-time quantitative polymerase chain reaction (RT-qPCR), and western blot analysis. Gastric sections for histological evaluation were stored in 10% buffered neutral formalin. The residue was frozen immediately in liquid nitrogen and stored at −80 °C.

### 2.4. Histological Analysis of Gastric Tissue

The individual gastric tissues (between cardiac and pylorus, the fundus) were sampled and fixed in 10% buffered neutral formalin for 24 h. We performed paraffin embedding, sectioning (3–4 μm in thickness), and staining with hematoxylin and eosin (H&E) for the general histopathology examination under a light microscope (Olympus PROVIS AX70; Tokyo, Japan) and NIS-Elements BR software (Nikon, Tokyo, Japan). For the histological index, the percentage of invaded lesions for each rat was calculated with the following formula (Equation (3)) [17,18]:

Percentage of invaded lesions (%) = (Length of lesions on the crossly trimmed fundic walls/total thickness of crossly trimmed fundic walls) × 100.(3)

### 2.5. Determination of Gastric Nitric Oxide

Gastric tissue homogenate was prepared in ice-cold PBS and then centrifuged at 5000× *g* for 5 min. The supernatant was used for the gastric NO measurement. The amount of NO in the stomach was determined with the Griess reagent system (G2930; Promega, Madison, WI, USA) according to the manufacturer’s instructions.

### 2.6. Measurement of Gastric Peroxidation

The determination of malondialdehyde (MDA) by thiobarbituric acid (TBA) was used as an index of the extent of lipid peroxidation [21,22]. Briefly, 0.1 g of stomach tissue was homogenized using 1 mL of 1.15% potassium chloride (Sigma-Aldrich, Co., St. Louis, MO, USA) followed by successive additions of 0.2 mL of 8.1% sodium dodecyl sulfate solution (SDS) (IBS-BS003a; iNtRON Biotechnology, Gyeonggi-do, Korea), 1.5 mL of 20% acetic acid (Daejung Chemical, Suwon, Korea), 1.5 mL of 0.8% aqueous TBA (Sigma-Aldrich, Co., St. Louis, MO, USA), and 0.7 mL of distilled water. The mixture was incubated in a water bath at 95 °C for 30 min followed by cooling on ice. We added 1 mL of distilled water and 5 mL of n-butanol (SAMCHUN, Gyeonggi-do, Korea) in a consecutive order. After mixing well, the mixture was centrifuged at 4000 rpm for 20 min (Combi-514R, Hanil Co. Ltd., Seoul, Korea). The absorbance of the clear supernatant was measured at 532 nm using an Epoch microplate spectrophotometer (BIOTEK, Inc., Winooski, VT, USA). The gastric MDA content was expressed as nmol MDA per g of stomach tissue. A standard curve was calculated with 1,1,3,3-tetraethoxypropane (Sigma-Aldrich, Co., St. Louis, MO, USA).

### 2.7. Determination of Gastric Antioxidant Enzyme

Gastric tissue was homogenized in ice-cold PBS and then centrifuged at 5000× *g* for 15 min. The gastric SOD, GPx, and CAT levels were measured using colorimetric assay kits (E02S0012, E02G0369, and E02C0086, respectively; BlueGene Biotech, Shanghai, China). The absorbance was read at 450 nm with a microplate reader.

### 2.8. Determination of the mRNA Expression in Gastric Tissue

The gastric tissue was homogenized with a homogenizer (Polytron PT 2500E, Kinematica AG, Luzern, Switzerland) and the total RNA was extracted using RNA extraction kit according to the manufacturer’s instructions (17221, iNtRON Biotechnology, Gyeonggi-do, Korea) and quantified with the spectrophotometer (BIOTEK, Inc., Winooski, VT, USA) at a 260/280 ratio. For the complementary DNA (cDNA) synthesis, the total RNA (50 ng/uL) was synthesized to the cDNA using a commercial kit (A2801, Promega, Madison, WI, USA) under the conditions of 25 °C for 5 min, 42 °C for 1 hr, 70 °C for 15 min, and 4 °C for 3 min using a thermal cycler (TP350, TaKaRa Bio, Otsu, Japan). To quantify the expressions of the genes, the cDNA, each primer, sterile purified water, and SYBR Green Master Mix (RR82LRB, TaKaRa Bio, Otsu, Japan) were mixed in a 96-well reaction plate (4316813, Thermo Fisher, Milford, MA, USA) according to the manufacturer’s instructions. After attaching the adhesive film (4311971, Thermo Fisher, Milford, MA, USA) to the plate, the reaction was then started using the real-time PCR machine (Applied Biosystems, Foster City, CA, USA) and the following conditions were maintained for 40 cycles (pre-denaturation at 95 °C for 3 min, denaturation at 95 °C for 10 s, annealing at 60 °C for 30 s, and extension at 72 °C for 30 s). The utilized PCR primers are shown in Table 1 and all gene expressions were normalized to the housekeeping gene, glyceraldehyde-3-phosphate dehydrogenase (*Gapdh*). All real-time PCR analysis was repeated three times.

### 2.9. Protein Extraction and Western Blot Analysis

The quantitation of gastric protein was carried out by western blot analysis. The gastric tissues were homogenized in Pro-prep reagent (17081; iNtRON Biotechnology, Gyeonggi-do, Korea) containing Halt^TM^ phosphatase inhibitor cocktails (Thermo Scientific, Waltham, MA, USA) and the protein concentrations were quantified using the PRO-MEASURE^TM^ kit (21011; iNtRON Biotechnology, Gyeonggi-do, Korea). Fifty micrograms of protein was resolved on 10% sodium dodecyl sulphate-polyacrylamide gel electrophoresis (SDS-PAGE) and subsequently transferred to a polyvinylidene difluoride (PVDF) membrane (Merck Millipore, Bedford, MA, USA) by electrophoretic transfer (Bio-Rad Laboratories, Inc., Hercules, CA, USA). 

The membranes were then blocked with 5% skimmed milk in tris buffered saline solution containing 0.1% Tween-20 (TBST) for one hour at room temperature. The membranes were washed three times with TBST and incubated with their respective primary antibodies at 4 °C overnight (Table 2), then washed again three times with TBST. Next, the membranes were incubated with the horseradish peroxidase-conjugated secondary antibody (Rabbit, W4011; Mouse, W4021; Promega, Madison, WI, USA) for one hour at room temperature in shaking conditions, followed by washing three times with TBST. The signals from the reaction with the enhanced chemiluminescence (ECL) western blotting detection reagent (16028; iNtRON Biotechnology, Gyeonggi-do, Korea) were visualized using an ImageQuant LAS 500 (GE Healthcare Life Sciences, Uppsala, Sweden).

### 2.10. Statistical Analyses

The statistical differences between the data for individual groups were assessed by one-way analysis of variance (ANOVA) with Duncan’s multiple range test using software SPSS version 23 (SPSS Inc., Chicago, IL, USA). A difference was considered statistically significant at *p* < 0.05. 

## 3. Results

### 3.1. Changes in the Body Weight of Rats

No significant difference in body weights were observed between the NC group and the HE group. Likewise, there were no significant differences in body weight changes between the experimental groups (FL 50, 100, and 200 mg/kg and ranitidine 30 mg/kg) and the HE group during the experimental period (Table 3).

### 3.2. Changes of Gastric Mucosa Gross Lesions

**Figure 1:**
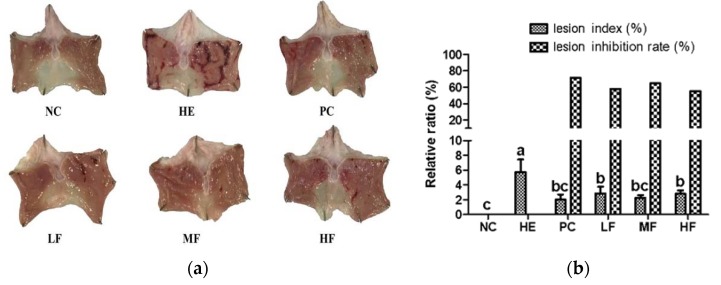
Effects of the fermented lotus root on the ulcer index in the EtOH/HCl-induced gastric ulcer model. (**a**) Macroscopic morphology changes in the gastric mucosa. (**b**) The lesion index (%) and lesion inhibition rate (%) of each group. The values are expressed as mean ± SD (n = 7 for each group). Different letters (a > b > c) indicate significant differences (*p* < 0.05) between the treatments determined by Duncan’s multiple range test. NC, normal control; HE, 150 mM HCl/60% EtOH control; PC, positive control; FL, fermented lotus root; LF, 50 mg/kg of FL; MF, 100 mg/kg of FL; HF, 200 mg/kg of FL.

Administration of EtOH/HCl mixture induced severe gastric damage. However, gastric damages were notably attenuated by pretreatment with FL or ranitidine (Figure 1a). The gastric lesion index (%) for the HE group accounted for 5.8% of the total area. However, in comparison to the HE group, significant (*p* < 0.05) reductions, i.e., 2.9%, 2.3%, 2.8%, and 2%, were observed in the FL groups and the PC group. In addition, the percent inhibition rates of stomach damage were significantly reduced in the FL groups (57.9%, 64.7%, 55.3%) and the PC group (71.4%), compared to the HE group (Figure 1b).

### 3.3. Microscopic Morphology of the Gastric Mucosa

**Figure 2:**
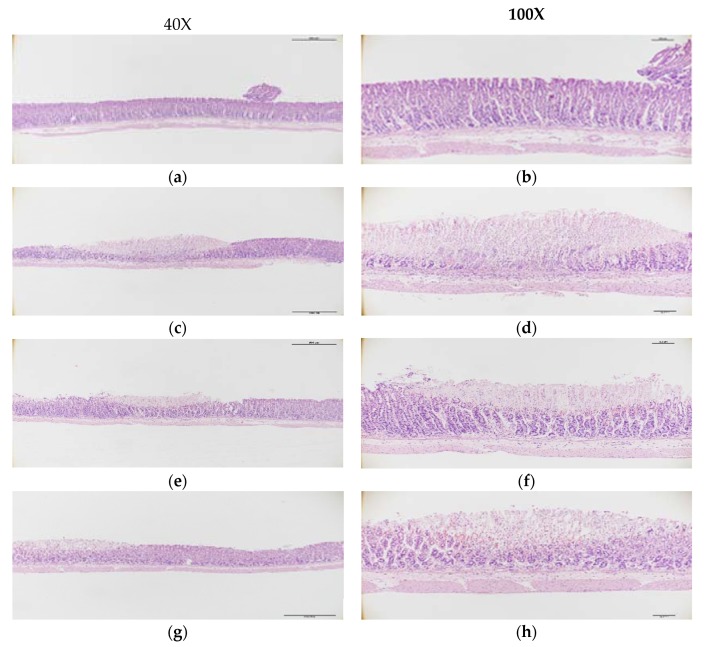

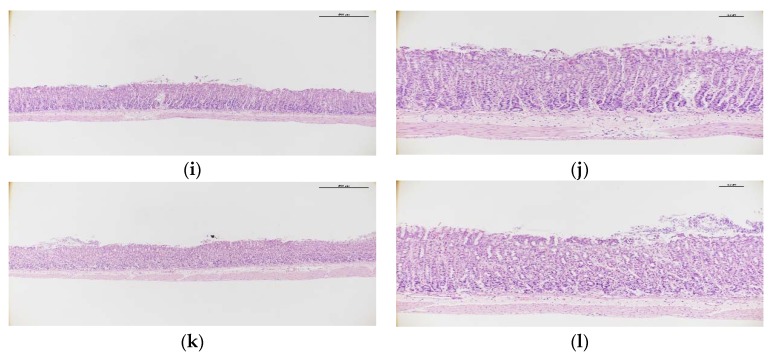
The effects of fermented lotus root on the histopathological lesions in the EtOH/HCl-induced gastric ulcer model. (**a**,**b**) NC, (**c**,**d**) HE, (**e**,**f**) PC, (**g**,**h**) LF, (**i**,**j**) MF, (**k**,**l**) HF.

H&E analysis showed that the gastric mucosal structure was within normal limits with no stomach glandular structural loss, hemorrhage, or submucosal edema in the NC group (Figure 2a). In contrast, extensive stomach glandular structural loss, edema, leukocyte infiltration, confluent necrosis, and serious hemorrhaging were observed in the HE group treated with EtOH/HCl (Figure 2b). Pre-treatment with ranitidine or FL resulted in an effective alleviation of these symptoms. In the PC group, the gastric mucosal structure was partially damaged. There was mild leukocyte infiltration and minimal hemorrhaging (Figure 2c). The LF group also displayed mild gastric mucosal structural loss and edema (Figure 2d). Meanwhile, the administration of a middle or high dose of FL effectively ameliorated the gastric injuries caused by the EtOH/HCl mixture. The MF group rarely displayed gastric mucosal necrosis, structural loss, or edema, but there was some leukocyte infiltration (Figure 2e). In the HF group, compared to the HE group that did induce severe gastric damage, there were not observed gastric mucosal necrosis, edema, hemorrhage, and leukocyte infiltration (Figure 2f).

**Figure 3:**
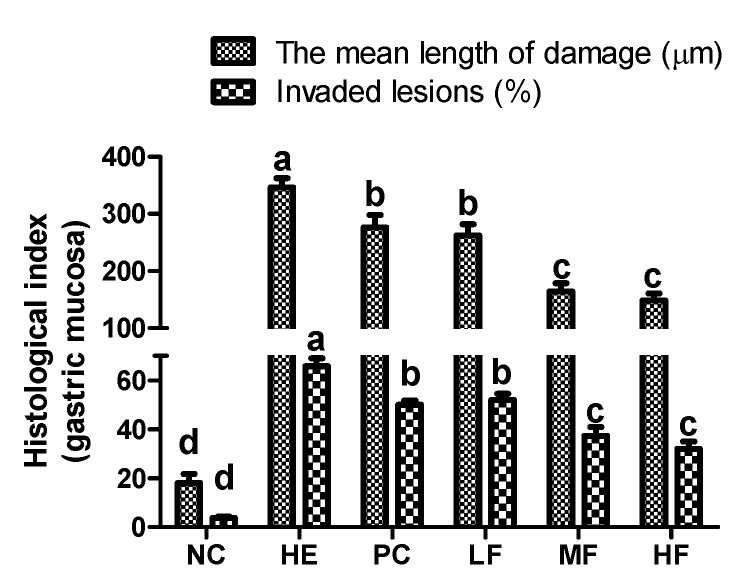
The effects of the fermented lotus root on the histopathological index of the gastric mucosa in the EtOH/HCl-induced gastric ulcer model. The values are expressed as mean ± SD (n = 3 for each group). Different letters (a > b > c > d) indicate significant differences (*p* < 0.05) between the treatments determined by Duncan’s multiple range test.

As shown in the results of the histomorphometric analysis (Figure 3), significant (*p* < 0.05) increases in the damaged gastric mucosa mean length and invasion percentages of lesions were observed in the HE group (346.2 ± 62.4 and 65.8%, respectively) compared to the NC group (18.1 ± 6.7% and 3.8%, respectively); however, these markers were significantly inhibited from elevation by the pre-treatment of FL (50, 100, and 200 mg/kg; 262.5 ± 48.0 and 52.0%, 164.1 ± 48.4 and 37.4%, and 148.5 ± 37.0 and 32.1%, respectively) or ranitidine (276.5 ± 67.5 and 50.0%, respectively). 

### 3.4. Effects of Fermented Lotus Root on Gastric NO and MDA

**Figure 4:**
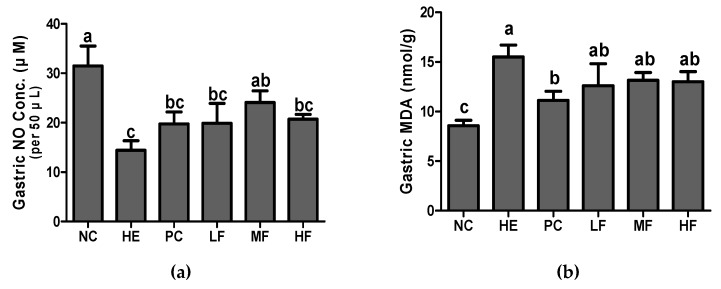
Effects of the fermented lotus root on the gastric nitric oxide (NO) and gastric malondialdehyde (MDA) in the EtOH/HCl-induced gastric ulcer model. (**a**) Gastric NO, (**b**) gastric MDA. Values are expressed as mean ± SD (n = 7 for each group). Different letters (a > b > c) indicate significant differences (*p* < 0.05) between treatments as determined by Duncan’s multiple range test.

As shown in Figure 4a, exposure to the EtOH/HCl mixture significantly decreased the gastric NO production and increased the gastric MDA in comparison to the NC group (*p* < 0.05). However, the pretreatment with FL or ranitidine markedly restored the reduction of NO production induced by the EtOH/HCl mixture (Figure 4a). The MDA levels in all FL groups showed a tendency to decrease in comparison to the HE group, although this was not significant (Figure 4b). 

### 3.5. Effects of Fermented Lotus Root on Antioxidant Defense System

**Figure 5:**
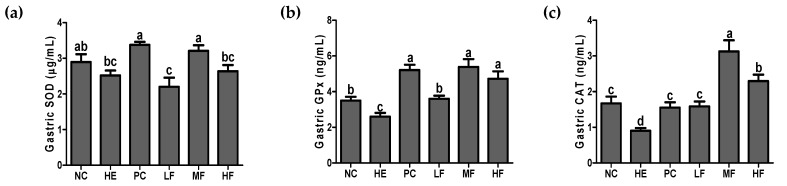
Effects of the fermented lotus root on the gastric superoxide dismutase (SOD), glutathione peroxidase GPx, and catalase (CAT) in the EtOH/HCl-induced gastric ulcer model. (**a**) Gastric SOD, (**b**) gastric GPx, and (**c**) gastric CAT. Values are expressed as mean ± SD (n = 7 for each group). Different letters (a > b > c > d) indicate significant differences (*p* < 0.05) between the treatments determined by Duncan’s multiple range test. SOD, Superoxide dismutase; GPx, glutathione peroxidase.

To evaluate antioxidant defense properties, concentrations of SOD, GPx, and CAT in gastric tissues were determined. Oral administration of the EtOH/HCl mixture resulted in a significant decrease in the antioxidant enzyme activities in the gastric tissue (Figure 5). As shown in Figure 5a, pre-treatment of middle dose of FL or ranitidine significantly increased the SOD activity compared with the HE group (*p* < 0.05). There was no significant difference in the gastric SOD activity between the HE and the low and high dose of FL group. In the gastric GPx and CAT results, the PC and all doses of FL groups showed significant increases compared to the HE group (Figure 5b,c, *p* < 0.05). Among them, administration of the middle dose (100 mg/kg) of FL exhibited a potent effect on the significant increase of gastric SOD, GPx, and CAT (*p* < 0.05).

### 3.6. Effects of the Fermented Lotus Root on Inflammation-Related mRNA Markers

**Figure 6:**
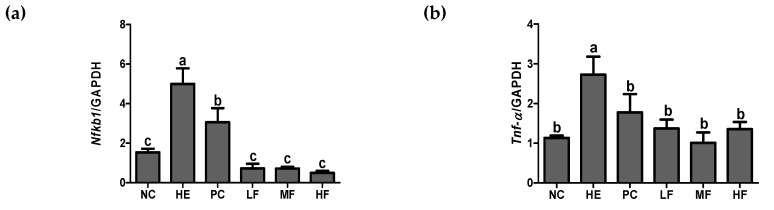

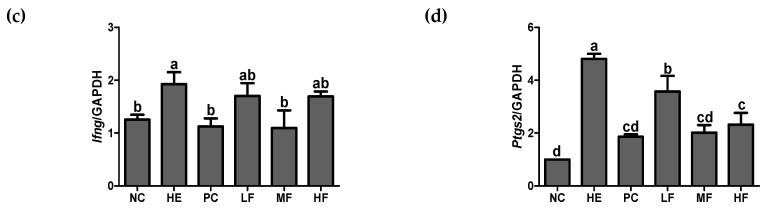
Effects of the fermented lotus root on the mRNA expression of gastric *Nfĸb1*, *Tnf-α*, *Ifng*, and *Ptgs2* in the EtOH/HCl-induced gastric ulcer model. (**a**) Gastric *Nfĸb1*, (**b**) gastric *Tnf-α*, (**c**) gastric *Ifng*, (**d**) gastric *Ptgs2*. The values are expressed as mean ± SD (n = 3 for each group). Different letters (a > b > c > d) indicate significant differences (*p* < 0.05) between the treatments determined by Duncan’s multiple range test. *Nfκb1*, nuclear factor kappa B subunit 1; *Tnf*, tumor necrosis factor; *Ifn*, interferon; *Ptgs2*, prostaglandin-endoperoxide synthase 2; *Gapdh*, glyceraldehyde-3-phosphate dehydrogenase.

To investigate the effects of FL on inflammation, we measured the mRNA expression of gastric inflammatory genes, *Nfĸb1*, *Tnf-α*, *Ifng*, and *Ptgs2* (Figure 6). In the HE group, the expression levels of *Nfĸb1*, *Tnf-α*, *Ifng*, and *Ptgs2* were significantly increased by tissue damage more than they were in the NC group (*p* < 0.05). However, the administration of FL or ranitidine exhibited a significant decrease in the expression of *Nfĸb1* and *Tnf-α* (*p* < 0.05). The mRNA expression of *Ifng* was significantly reduced in the PC group and in the MF group (*p* < 0.05). In the LF and MF group showed a tendency to decrease in comparison to HE group, although this was not significant. The mRNA expression of *Ptgs2* was significantly decreased in the PC and FL groups compared with the HE group (*p* < 0.05).

### 3.7. Effects of Fermented Lotus Root on NF-ĸB Pathway

**Figure 7:**
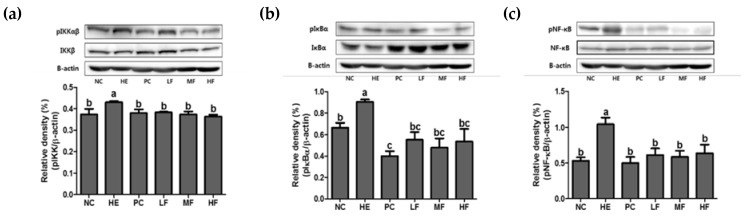
The effects of the fermented lotus root on the protein expression of the NF-κB signaling pathway in the gastric tissue of EtOH/HCl-induced gastric ulcer model. (**a**) Gastric pIKKαβ/IKKβ, (**b**) gastric pIκBα/IκBα, (**c**) gastric pNF-κB/NF-κB. Values are expressed as mean ± SD (*n* = 3 for each group). Different letters (a > b > c) indicate significant differences (*p* < 0.05) between treatments as determined by Duncan’s multiple range test. IκB, inhibitor of κB; IKK, IκB kinase; NF-κB, nuclear factor κB.

To verify the mechanisms of the gastroprotective effects of FL on the gastric ulcer model, the protein expressions related to the inflammation-signaling pathways were evaluated (Figure 7). As shown in Figure 7, the EtOH/HCl mixture treatment significantly elevated the phosphorylation expressions of IKK, IκBα, and NF-κB in gastric tissue. However, oral-administration of FL or ranitidine at all doses significantly restored the levels of IKK, IκBα, and NF-κB phosphorylation (*p* < 0.05).

## 4. Discussion

Gastric ulcers are one of the most common diseases. They occur due to an imbalance between the aggressive gastric factors and defensive mucosal factors. Alcohol is a powerful risk factor for the gastric mucosa. Ethanol intake directly damages the vascular endothelial cells of the gastric mucosa and causes excessive production of oxygen radicals through an inflammatory cascade [23]. Furthermore, ROS can act as second messengers to trigger several redox-sensitive signaling transduction cascades, such as NF-κB and mitogen-activated protein kinases (MAPKs). 

The NF-κB signaling pathway is closely linked to the inflammatory state of the stomach [24]. Modulation of the NF-κB signaling is considered to be a therapeutic target in stomach diseases [25], and fermented lotus root may provide a novel therapeutic candidate. The obtained data in the present study demonstrated the gastroprotective effects of fermented lotus root against gastric ulcers induced by an EtOH/HCl mixture in rats. The underlying mechanism was associated with antioxidant and anti-inflammatory properties through the NF-κB signaling pathway.

Ethanol leads to severe damage in the gastric mucosa as ethanol penetrates easily into the gastric mucosa [25]. Our results confirmed that administration of the EtOH/HCl mixture induced severe macroscopic gastric damage. Ulceration and patchy hemorrhages were also clearly observed by microscopy. However, oral administration of ranitidine or FL markedly alleviated the gastric damage induced by the EtOH/HCl mixture. The PC and LF groups were shown by microscopic observation to have less damaged mucosa than those of the HE group, and the MF and HF groups were observed to have almost normal surface epithelial cells. The mechanism for ulcer repair is a process that regards the balance of cell damage and repair at the ulcer site [1]. Our results demonstrated that the administration of FL before stomach injury reduced the gastric wall pathogenicity and recovered the damage to a level similar to that of the NC group.

In the gastrointestinal (GI) tract, NO has a dual function at the gastric mucosal level [26]. One of these functions is the endothelial nitric oxide synthase (eNOS) that produces NO to assist in gastric ulcer healing mainly through stimulation of the vascular integrity and mucus production, increasing the blood flow, and anti-inflammatory action [27], while excessive NO generated from inducible nitric oxide synthase (iNOS) was involved in the tissue damage via the formation of ROS and the toxic effects on cells [28]. Maintaining normal levels of NO is very important in stomach health as this inhibits neutrophil infiltration and helps blood circulation as a vasodilator in the stomach [26]. Therefore, the normal production of NO can retain the integrity of the gastric mucosa and contribute to the defense and healing of mucosal damage. The results of the present study also demonstrated that administration of FL pre-treatment led to increased gastric NO production.

Acute gastritis is closely associated with oxidative stress and inflammation, including neutrophil infiltration [27]. Excessive oxidative stress causes excess cellular levels of ROS. As a result, these ROS cause injury to normal cells and tissues and induce the production of toxic products, such as free radicals [28]. The level of MDA is considered an important biomarker that reflects free radical-mediated membrane damage. In this study, the MDA content was significantly increased after gastric injury in the HE group compared with the NC group. However, pre-treatment with ranitidine significantly alleviated the EtOH/HCl-induced MDA increase in the stomach. FL administrations showed a non-significant reduction in the MDA content, but a decreased tendency was observed compared with those of the HE group.

The state of excessive oxidative stress results in the overproduction of ROS, which devastates the endogenous antioxidant capacity. Our results demonstrated that acute gastric injury induced the oxidative stress status in the gastric mucosa and depleted antioxidant enzyme activities, such as SOD, CAT, and GPx. However, the administration of ranitidine or FL improved the antioxidant defense system in the stomachs of gastric damaged rats. These outcomes can be partially explained by the ability of the ranitidine and FL to reduce the MDA levels. Antioxidants are molecules that inhibit or quench free radical reactions and delay or inhibit cellular damage in the stomach [29]. Phenolics and flavonoids are the most important plant secondary metabolites, which display robust pharmaceutical activities including antioxidant capacity [30]. *N. nucifera* has been reported to produce important secondary metabolites, including alkaloids, flavonoids, steroids, triterpenoids, glycosides, and polyphenols [14]. Therefore, these results indicate that the high antioxidant potential of FL increased the activity of antioxidant enzymes in the stomach for its protection. 

ROS overproduction activated the NF-κB signaling pathway, which is the principal transcription factor of hundreds of genes, such as the cytokines/chemokines that are involved in regulating cell growth, differentiation, development, and apoptosis [8]. The involvement of inflammatory mediators in EtOH/HCl-induced gastric ulcers was previously reported [18,31,32]. In a state of inflammation, the pro-inflammatory mediators, such as TNF-α, inter leukin (IL)-1, and IL-6 stimulated and activated NF-κB, which, in turn, activated the subunits of the IKK complex. IKK is composed of catalytic (IKKα and IKKβ) and regulatory (IKKγ; also known as NF-kappa-B essential modulator) subunits. 

Consequently, activated IκB undergo ubiquitylation and proteasomal degradation, and the freed NF-κB is translocated to the nucleus. This step is critical in the acceleration of the inflammatory response [9]. In our current work, pre-treatment with ranitidine or FL before gastric injury remarkably reduced the gastric *Nfκb1*, *Tnf-α*, *Ifng*, and *Ptgs2* mRNA expression levels. In addition, the NF-κB pathway related protein marker (gastric pIKKαβ/IKKβ, pIκBα/IκBα, and pNF-κB/NF-κB) expressions were significantly improved following ranitidine or FL supplementation. These results indicated that the mechanism of the FL ameliorating gastric ulcer might be associated with its anti-inflammatory effect through modulating the NF-κB signaling pathway.

Taken together, our findings demonstrated that FL pre-treatment exerted beneficial effects in EtOH/HCl-induced acute gastric ulcers. Further studies are warranted to determine whether the same effect occurs in normal (non-fermented) lotus root and to explore the clinical application of the FL. 

## 5. Conclusions

In the present study, our results revealed that administration of FL significantly prevented gastric injury through elevation of the antioxidant enzyme activities and alleviation of inflammatory conditions by inhibiting the NF-κB pathway in gastric damage-induced EtOH/HCl stimulation. These findings suggested that fermented lotus root was appropriate as a medicinal natural food for gastric ulcer therapy to regulate the antioxidant enzymes and anti-inflammatory related genes.

## Figures and Tables

**Table 1:** List of the sequences of the primers used for reverse transcription real-time quantitative polymerase chain reaction (RT-qPCR).

Genes	Forward Sequence (5′-3′)	Reverse Sequence (3′-5′)
*Nfkb1*	GACCCAAGGACATGGTGGTT	TCATCCGTGCTTCCAGTGTTT
*Tnf-α*	GTGATCGGTCCCAACAAGGA	CTCCCACCCTACTTTGCTTGTG
*Ifng*	GATCCAGCACAAAGCTGTCA	GACTCCTTTTCCGCTTCCTT
*Ptgs2*	CCAGCAGGCTCATACTGATAGGA	GCAGGTCTGGGTCGAACTTG
*Gapdh*	TGGTGAAGGTCGGTGTGAAC	TTCCCATTCTCAGCCTTGAC

*Nfκb1*, nuclear factor kappa B subunit 1; *Tnf*, tumor necrosis factor; *Ifn*, interferon; *Ptgs2*, prostaglandin-endoperoxide synthase 2; *Gapdh*, glyceraldehyde-3-phosphate dehydrogenase.

**Table 2:** List of the primary antibodies we used in the western blotting analysis.

Antigen	Source	Dilution	Manufacturer	Catalog Number
Phospho-IKKα/β	Rabbit	1:1000	Cell signaling technology	2697
IKKβ	Rabbit	1:1000	Cell signaling technology	8943
Phospho-IκBα	Rabbit	1:1000	Cell signaling technology	2859
IκBα	Mouse	1:1000	Cell signaling technology	4814
Phospho-NF-κB p65	Rabbit	1:1000	Cell signaling technology	3033
NF-κB p65	Rabbit	1:1000	Cell signaling technology	8242
β-actin	Mouse	1:10,000	Abcam	ab6276

Phospho, phosphorylation; IκB, inhibitor of κB; IKK, IκB kinase; NF-κB, nuclear factor κB; β-actin, beta-actin.

**Table 3:** Changes in the body weight gains of each group (g).

Groups	Initial Body Weight (A)	1 Week	2 Weeks	Terminal Body Weight (B)	Body Weight Gains (B–A)
**Controls**					
NC	241.7 ± 21.1 ^ns^	278.9 ± 13.1 ^ns^	312.7 ± 17.2 ^ns^	341.8 ± 21.9 ^ns^	100.2 ± 16.6 ^ns^
HE	241.9 ± 18.9	278.3 ± 11.9	313.5 ± 13.7	349.6 ± 24.2	107.8 ± 22.5
**Reference**					
PC	241.8 ± 18.7	278 ± 11.3	316.6 ± 14.1	348 ± 16.8	106.2 ± 14.0
**Experiments**					
LF	241.7 ± 18.6	276.2 ± 9.1	313 ± 10.8	342.3 ± 15.3	100.7 ± 25.7
MF	243.8 ± 12.6	279.3 ± 11.0	317.6 ± 15.8	348.1 ± 19.6	104.3 ± 18.8
HF	244.2 ± 12.1	280 ± 8.5	320.4 ± 10.1	352.9 ± 13.4	108.7 ± 9.8

Values are expressed as mean ± S.D (*n* = 7 for each group). NC, normal control; HE, gastric ulcer model control, 150 mM HCl/60% EtOH control; PC, positive control; FL, fermented lotus root; LF, 50 mg/kg of FL; MF, 100 mg/kg of FL; HF, 200 mg/kg of FL; ns, not significant.
